# Acute Ulcerative Gingivitis

**Published:** 1919

**Authors:** 


***Acute Ulcerative Gingivitis.*** By CLAUD G. COLYER. *British Medical Journal*, Oct. 12, 1918.

*Signs and Symptoms.* The general symptoms are as follows: malaise; slight elevation of temperature, rarely over 100 F. but occasionally 102 F.; glandular swelling, the submaxillary glands being chiefly affected; general depression.

The local symptoms are: hemorrhage from the gums, either spontaneous or as the result of pressure; pain in the gums especially at night, and often so severe as to make sleep impossible; a foul taste; very offensive breath; mastication difficult and extremey painful; the teeth becoming very tender and loose.

*Clinical History.* The onset is gradual.

*Local Signs.* In the earlier stages the only signs are a slight edema of the inter-dental papillae. Later the gums appear to be separated from the teeth, this being due to the swelling and not to any destruction of the periodontal membrane. An acute necrosis of the papillae then follows. If left untreated, sloughing gradually spreads until a considerable area of gum is destroyed. The gums in the infected regions are deeply congested. The teeth are usually loose.

*Distribution of the Lesion.* The gums around the upper incisor teeth are commonly affected first. When one jaw is affected the other soon shows signs of affection in a corresponding situation.

*Pathology and Bacteriology.* In the early stages the tissues attacked by the specific organism become deeply congested, and after two or three days necrose, making a series of cup-shaped depressions between the teeth. Tartar is always present on the teeth exposed by the sloughing of the gums.

The characteristic organisms found in films are a mixture of bacillus fusiformis and spirilla.

*Incidents and Sources of Infection.* Men in the trenches or those living under bad conditions are only slightly more liable to infection than those under more favorable circumstances. The condition is more prevalent during the winter than during the summer months.

Up to the present the author has not been able to trace any definite methods of infection.

Excessive smoking in his opinion predisposes to the condition, and considerably retards the process of healing. The absence of fresh food does not seem to play any part in the causation. Officers and men who are able to obtain fresh vegetables are frequently victims.

*Duration.* If untreated, the condition becomes chronic and results, in a year or eighteen months, in the destruction of the bony sockets of the teeth. When this occurs, the teeth must necessarily be extracted before a cure can be effected. If treated vigorously all symptoms disappear in three or four days, although a cure is not effected until about the tenth day of treatment; even after this period films may still show the presence of the causative organisms. Careful use of a tooth brush and the employment of an astringent application to the gums for two or three weeks as an after-treatment will tend to decrease these recurrences.

*Diagnosis.* In the earlier stages diagnosis may be difficult. Suspicion may be aroused by a slight swelling and reddening of an isolated interdental papilla, the surface of which appears to be stretched and glossy, and the edges thinned out and transparent. At this stage a microscopical examination should be made to establish the diagnosis. In the later stages the characteristic bluish red gum covered by slough, the offensive breath and the glandular enlargement make the diagnosis easy.

*Treatment.* The treatment adopted by the author as a result of considerable experience is briefly as follows :

*General Treatment.* Diet is light for the first two days. Smoking is forbidden.

*Local Treatment.* All slough is scraped away. The remaining debris is washed away with a concentrated solution of thymol in water. If thymol is not available eusol may be used instead. The gums are then dried with a five per cent. solution of silver nitrate, after which the mouth is washed out with water or thymol solution.

*Subsequent Treatment.* A mouth wash of thymol water is given two-hourly throughout the day, until all ulcers have a clean granulating surface. All roots and septic teeth are then extracted and all necessary fillings completed as soon as the ulcers commence to heal.

Glycerin and tannic acid are rubbed into the gums daily when the application of silver nitrate has been discontinued.

*Treatment of Chronic Cases.* This is different from above in that any attempt to save the teeth in the affected areas is useless.

The usual duration of the disease from the commencement of treatment is from ten to fourteen days, patients usually becoming free from the specific organism on the eighth or ninth day.

Figures show that acute ulcerative gingivitis is present in approximately 0.3 per cent. of men behind the lines and in 0.7 per cent. of those returning from the trenches. Severe cases represent 0.63 per cent. of all men reporting sick on account of their teeth.

